# Lung Troubles after Anæsthetics

**Published:** 1908-07-11

**Authors:** 


### Anæsthetics.

### LUNG TROUBLES AFTER ANÆSTHETICS.

Dr. Otis B. Wight, a former house officer in the Johns Hopkins Hospital, has written a most interesting and instructive account of the pulmonary complications that follow anæsthesia, in the "Johns Hopkins Hospital Bulletin." He bases his remarks upon an analysis of 9,000 cases anæsthetised in the gynæcological wards, so that his statistics apply to adults. It would be tedious to enter into lists of figures; for those the original paper may be consulted. It is rather with the general conclusions that we would deal.

A greater number of the post-operative pneumonias are lobular rather than lobar in type; nevertheless the lobar type is not absolutely uncommon. The second or third day after the operation is the most usual time for the development of the symptoms. The latter consist of a shivering attack or rigor, pain in the affected side of the chest, cough, and pyrexia. The right lung is affected more often than the left, but the distribution in the right lung is variable, the upper part being almost as frequently involved as the lower. This is the reverse of what would be expected if the trouble were due to inhalation of septic particles from the mouth. There is no marked liability for empyema or abscess of the lung to result, but the mortality is about the same as it is for lobar pneumonia due to the pneumococcus in adults generally—namely, about 33 per cent. of the affected cases.

The lobar pneumonia which follows anæsthesia presents certain clinical points of distinction from ordinary lobar pneumonia: (1) The pyrexia tends to be less marked and less maintained. The patient's temperature rarely reaches 103° F., and the curve is more irregular and not so steady as it is in typical pneumonia; (2) the duration is usually shorter; (3) there is rarely an obvious crisis; (4) complications such as empyema are exceptional; (5) the physical signs of the lung trouble are often ill-marked. The consolidation seems to be central rather than at the surface of the lung, so that it may be several days before abnormal signs can be detected at all; slight impairment of percussion note, with a few crepitant râles, associated with pyrexia and a quickened pulse, may be all that can be detected as indications of the pneumonia. Bronchial breathing and marked impairment of the percussion note may not be observed at all, or only when the trouble has existed for some time and the temperature is already beginning to fall; (6) cough is neither marked nor severe as a rule, and the sputum is seldom blood tinged or rusty.

The duration of the anæsthesia plays a considerable part in the production of the pulmonary complications. In Dr. Wight's series, out of all the cases that were under the anæsthetic for one hour or less only four developed pneumonia; whereas eleven patients suffered from the latter when the anæsthesia had been prolonged for two hours or more. The position of the patient in bed after laparotomy has also an immense influence. Until recent years it has been customary to keep the patient lying flat. It is now the rule to adopt the semi-sitting posture as soon as the immediate results of the anæsthetic have passed off—this posture being maintained by means of props and pillows without the least exertion on the patient's part. This procedure is now generally adopted in England, and the benefits of it are obvious, particularly in cases of general peritonitis.

Wound healing is not materially affected by the development of pneumonia. This is in accord with the general view of all writers upon the subject.

It is not at all easy to distinguish with certainty between broncho-pneumonia and lobar pneumonia after operations, although some observers try to separate even a third group of cases, to which they give the name hypostatic pneumonia. The latter is chiefly characterised by the development of small patches of pneumonia at the bases of the lungs in elderly people and in patients seriously weakened by disease. If hypostatic and broncho-pneumonia be classed together, 17 of Dr. Wight's cases developed it and two died, giving a mortality of 12 per cent.

The trouble was very similar to that of lobar pneumonia, except in duration. It generally appeared from one to three days after the operation, and lasted from 7 to 12 days in the cases that recovered. The onset was by chill and rise of temperature, the latter being considerably higher in the evening than in the morning, and not following the usual curve that the temperature does in lobar pneumonia. Cough was not a marked feature, and cyanosis and very rapid respirations were rarely noticed.

The physical signs usually consisted in mucous râles throughout both lungs, especially in the lower lobes; over small areas of lung tissue bronchial breathing might be heard, and some impairment of percussion note could be detected. This impairment of note was often not discernible until shortly before the temperature began to drop.

A true fibrinous or serous pleurisy unassociated with pneumonia is one of the rarer complications of anæsthesia. Only a small number of the patients who actually have pneumonia develop the characteristic creaking or grating sounds of pleuritic friction. The few cases of fibrinous pleurisy that did occur had the typical symptoms: sharp stabbing pain in the side, dry painful cough, and moderate pyrexia. These disappeared after a few days, and the healing of the wound was not retarded. A smaller but unknown percentage of these patients develop an effusion with the typical physical signs. In no case was paracentesis thoracis needed. Pleurisy developed in nearly every instance within the first 48 hours; no case proved fatal.

Thirty cases suffered from bronchitis simply, and all of them recovered. The symptoms and signs were chiefly a feeling of tightness in the chest, slight pyrexia, and squeaking rhonchi or non-consonating râles, mainly found at the bases of the lungs without impairment of the percussion note. The patients so affected were at no time seriously ill, and the signs and symptoms cleared up promptly without retarding convalescence.

In order to minimise the risk of post-operative lung complications, particularly in the weakly or in the old, disinfection of the mouth and nose by mild antiseptic gargles and douches should be a routine measure to be adopted and not only just before the operation, but also for a day or two before it wherever feasible.

Careful administration of the anæsthetic, preferably by the drop method on an open mask, given slowly and steadily at first so that the patient is not suffocated and does not struggle, is important. The head should be low, and kept upon one side, so that mucus flows outwards instead of down the throat. Much more care than is the rule should be observed in trying to keep the patients warm and dry whilst they are upon the table; and, when the operation is over, the patient should be warmly covered up whilst being carried back to bed.

